# Artificial intelligence in rheumatology: A transformative perspective

**DOI:** 10.1515/jtim-2025-0046

**Published:** 2025-10-16

**Authors:** Lusi Ye, Xin Li, Hamzeh Ghorbani, Xiaofei Zhou, Arsen Minasyan, Bolun Zheng, Xiaotian Pan, Guodao Zhang, Dan Chen

**Affiliations:** Department of Rheumatology, The First Affiliated Hospital of Wenzhou Medical University, Wenzhou, Zhejiang Province, China; Institute of Intelligent Media Computing, Hangzhou Dianzi University, Hangzhou, Zhejiang Province, China; Shangyu Institute of Science and Engineering Co.Ltd., Hangzhou Dianzi University, Shaoxing, Zhejiang Province, China; Faculty of General Medicine, University of Traditional Medicine of Armenia, Yerevan, Armenia; School of Automation, Hangzhou Dianzi University, Hangzhou, Zhejiang Province, China

## Introduction

Rheumatology, a specialty focused on autoimmune and musculoskeletal disorders, faces persistent diagnostic challenges that hinder timely and effective patient care. In recent years, artificial intelligence (AI), particularly through advancements in machine learning (ML), has emerged as a transformative force across healthcare. By enabling the analysis of complex, multimodal datasets, AI offers unprecedented opportunities to enhance diagnostic precision, personalize treatments, and optimize outcomes for patients with rheumatic diseases. Real-world applications of AI-powered tools are already demonstrating their impact, offering new pathways for earlier detection and more precise treatment strategies. However, their integration into clinical practice also raises important ethical and practical considerations. As AI continues to transform the healthcare landscape, its influence in reshaping rheumatology is becoming increasingly evident—paving the way for more accurate diagnoses, tailored therapies, and enhanced patient care experiences.^[1, 2, 3]^

**Figure 1:**
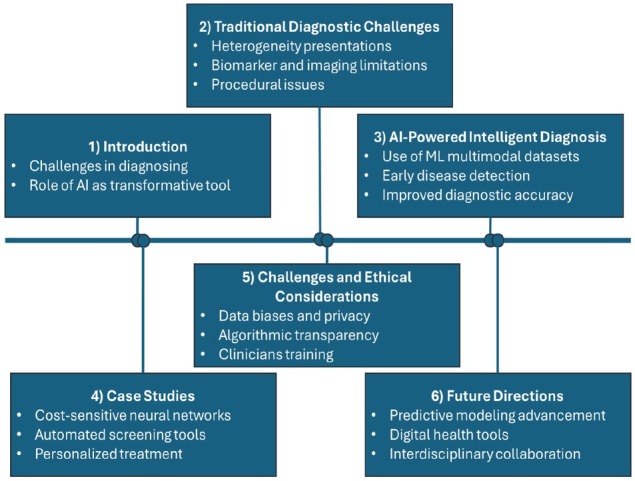
Flowchart of artificial intelligence in rheumatology: A transformative perspective.

Figure 1 illustrates the flowchart for the present article “Artificial intelligence in rheumatology: A transformative perspective”, which systematically examines the integration of AI into rheumatology to address diagnostic challenges and enhance patient care. The flowchart is divided into six key sections: introduction, traditional diagnostic challenges, AI-powered intelligent diagnosis, case studies, challenges and ethical considerations, and future directions. The Introduction section highlights the persistent challenges in diagnosing rheumatic diseases and introduces AI as a groundbreaking tool to address these inefficiencies. The traditional diagnostic challenges section outlines the complexity of clinical presentations, the limitations of biomarkers and imaging techniques, and the procedural constraints that often delay accurate diagnoses. Moving forward, the AI-powered intelligent diagnosis section highlights the capabilities of machine learning algorithms to analyze complex, multimodal datasets and detect early disease patterns, thereby improving diagnostic precision and efficiency. The case studies section showcases real-world applications of AI, including cost-sensitive neural networks that enhance diagnostic accuracy, automated screening tools that expand access to care in underserved areas, and personalized treatment strategies informed by predictive modeling. The challenges and ethical considerations section critically examines data bias, privacy risks, and the necessity of transparent algorithms and clinician education to ensure the ethical and effective deployment of AI. Lastly, the future directions section envisions advancements in predictive modeling, the proliferation of digital health tools, and the importance of interdisciplinary collaboration among healthcare providers, data scientists, and policymakers.

## Traditional diagnostic challenges in rheumatology

The accurate and timely diagnosis of rheumatological diseases remains a significant challenge due to the inherent complexity of autoimmune and musculoskeletal conditions. Traditional diagnostic methods rely on clinical expertise, laboratory testing, and imaging techniques, each with its critical limitations, which contribute to delays and variability in care. Diagnosis in rheumatology is highly dependent on the expertise of individual clinicians, resulting in substantial interobserver variability. Conditions such as rheumatoid arthritis (RA) often present with subtle and overlapping symptoms, making early recognition particularly difficult. For example, identifying synovitis—a hallmark of RA—requires advanced clinical acumen, and inconsistent recognition can lead to delayed interventions and suboptimal management strategies.^[4]^

Commonly used biomarkers, such as rheumatoid factor (RF) and anti-cyclic citrullinated peptide (anti-CCP) antibodies, are imperfect tools for diagnosis. RF is present not only in RA but also in other autoimmune diseases and even in healthy individuals, resulting in false positives. Similarly, anti-CCP, while more specific, lacks complete sensitivity, particularly in early-stage RA. These limitations can lead to diagnostic uncertainty, delayed therapy initiation, and missed opportunities for early intervention.^[5]^ Although essential in detecting joint pathology, imaging modalities are limited in their capabilities. X-rays often fail to detect early joint damage or subtle erosions, while ultrasound is operator-dependent, requiring substantial expertise to ensure diagnostic accuracy. Though more precise, magnetic resonance imaging (MRI) is expensive, time-intensive, and impractical for routine use in many healthcare settings. These challenges reduce the utility of imaging as a standalone diagnostic tool in routine practice.^[6]^

Procedures such as synovial biopsies and arthrocentesis can provide valuable diagnostic insights but are often associated with patient discomfort and risk of complications. For instance, synovial biopsy, although informative, involves risks such as bleeding and infection, leading to patient reluctance. Similarly, arthrocentesis, while helpful in diagnosing synovial inflammation, may be avoided due to pain and the potential for post-procedure joint infections.^[7]^ The cumulative effect of these challenges results in prolonged diagnostic timelines, increased healthcare costs, and a reduced quality of life for patients.

## AI-Powered intelligent-assisted diagnosis

ML algorithms have emerged as powerful tools for enhancing the diagnosis of rheumatological diseases. These algorithms leverage computational techniques to analyze complex datasets and identify patterns that may not be readily apparent to human clinicians. In rheumatology, ML algorithms offer several advantages for improving diagnostic accuracy and efficiency. One of the key strengths of ML algorithms is their ability to recognize patterns in multimodal data, including clinical assessments, laboratory results, and imaging studies. By integrating diverse information sources, ML algorithms can generate comprehensive diagnostic models that capture the multifaceted nature of rheumatological diseases. These models can guide further diagnostic evaluations and treatment decisions, leading to more accurate and timely diagnoses. ML algorithms detect subtle patterns in data, making them particularly well-suited for early disease detection. In rheumatology, early diagnosis is critical for initiating appropriate treatment and preventing irreversible joint damage. ML algorithms can analyze imaging studies, such as X-rays and MRI scans, to identify early signs of inflammation and joint damage that may not be apparent to human observers. By detecting these changes at the earliest stages of the disease, ML algorithms can facilitate prompt intervention and improve patient outcomes.^[8, 9, 10]^

## Challenges and ethical considerations

Despite its transformative potential, the implementation of AI in rheumatology faces significant challenges, particularly in terms of data quality, biases, and ethical considerations. AI algorithms rely on diverse, high-quality datasets to ensure accurate predictions, yet many healthcare datasets are incomplete, heterogeneous, or biased. These limitations can skew AI models, exacerbating healthcare disparities, especially for underrepresented populations. Ethical concerns also arise regarding data privacy, security, and transparency. Using sensitive patient data in AI systems poses risks of breaches and misuse, requiring stringent safeguards to protect confidentiality.

## Future directions and conclusion

AI is poised to revolutionize rheumatology by enhancing diagnostic accuracy, personalizing treatments, and empowering patients through digital innovations. Predictive modeling using AI can identify novel biomarkers and forecast disease trajectories, enabling earlier and more targeted interventions. Digital health tools, including mobile apps and wearables, promote patient engagement and adherence, fostering a proactive approach to disease management. Importantly, the integration of privacy-preserving technologies, such as federated learning, holds great promise in enabling secure multi-center collaborations without compromising sensitive patient data. These approaches can help build robust, generalizable AI models by aggregating insights across institutions while maintaining strict data confidentiality and privacy.

Interdisciplinary collaboration among rheumatologists, data scientists, and policymakers is crucial to ensure the ethical and practical integration of AI technologies into clinical practice. Equally important is the establishment of structured training frameworks to support clinicians in adopting AI tools. Educational initiatives should focus on developing literacy in data interpretation, algorithmic transparency, and limitations of AI predictions. Moreover, real-world evaluation metrics such as clinical utility, patient outcomes, and system-level performance must be standardized to monitor and continuously improve AI effectiveness in daily clinical settings. Addressing current barriers, such as data quality and algorithmic transparency, AI can transform rheumatology into a more precise, equitable, and patient-centered field, ultimately improving outcomes and satisfaction. As advancements continue, AI-driven rheumatology promises to revolutionize care delivery, achieving unprecedented levels of personalization, prevention, and efficiency.
